# Self-Referential Processing and Resting-State Functional MRI Connectivity of Cortical Midline Structures in Glioma Patients

**DOI:** 10.3390/brainsci12111463

**Published:** 2022-10-28

**Authors:** Chuh-Hyoun Na, Kerstin Jütten, Saskia Doreen Forster, Hans Clusmann, Verena Mainz

**Affiliations:** 1 Department of Neurosurgery, RWTH Aachen University, 52074 Aachen, Germany; 2 Center for Integrated Oncology Aachen Bonn Cologne Duesseldorf (CIO ABCD), 52074 Aachen, Germany; 3 Institute of Medical Psychology and Medical Sociology, RWTH Aachen University, 52074 Aachen, Germany

**Keywords:** default mode network, self-referential processing, cortical midline structures, trait judgement task, trait recall task, resting-state functional MRI, glioma

## Abstract

Metacognition has only scarcely been investigated in brain tumor patients. It is unclear if and how the tumor-lesioned brain might be able to maintain an adequate sense-of-self. As cortical midline structures (CMS) are regarded as essential for self-referential mental activity, we investigated resting-state fMRI connectivity (FC) of CMS to the default-mode network (DMN) and to the whole brain, comparing glioma patients and matched controls. Subjects furthermore performed a trait judgement (TJ), a trait recall task (TR), and neuropsychological testing. In the TJ, adjectives had to be ascribed as self- or non-self-describing, assessing the self-serving effect (SSE), a normally observed bias for positive traits. In the TR, the mnemic neglect effect (MNE), a memory advantage for positive traits, was tested. The groups were compared and partial correlations between FC and test metrics were analyzed. Although patients were significantly impaired in terms of verbal memory, groups did not differ in the SSE or the MNE results, showing preserved metacognitive abilities in patients. FC of CMS to the DMN was maintained, but was significantly decreased to whole brain in the patients. FC of the dorsomedial prefrontal cortex (DMPFC) to whole brain was correlated with the MNE in patients. Preserving the DMPFC in therapeutic interventions might be relevant for maintaining self-related verbal information processing in the memory domain in glioma patients.

## 1. Introduction

While cognitive dysfunction is commonly observed in glioma patients across different cognitive domains [1,2,3,4,5,6], metacognitive abilities such as self-referential processing have only scarcely been investigated in brain tumor patients. With ‘the self’ being a multidimensional construct vaguely conceptualized by philosophical, psychological, or neurobiological accounts [7,8,9,10,11,12], it is, however, a prerequisite for the formation of a first-person perspective and fundamental to personality, self-awareness, and ultimately consciousness. While some brain tumor patients may show not only cognitive impairment but changes in affect, social behavior, and personality, others seem to be unaffected in this regard. It is, however, unclear to what extent and depending on which neural correlates the tumor-lesioned brain might be able to maintain an adequate sense of self.

One way to scientifically approach ‘the self’ is to investigate self-referential processing (SRP), denoting the ability to relate external stimuli, memories, thoughts, or emotions to oneself. Intact SRP has to be considered as essential for the formation of a stable self-concept and the ability for self-reflection as a premise to adequately interact socially. Disturbances in SRP on the other hand have repeatedly been related to psychological and social dysfunction in various neurological and psychiatric disorders [13,14,15,16,17,18,19], impairing functional and emotional well-being as well as sociocognitive integration. Alterations in SRP in brain tumor patients may impact on self-perception and may alter insights into one´s own condition, potentially leading to maladaption and impacting on disease coping.

SRP differs from non-self-related information processing in healthy subjects, in that self-related information is normally biased. A well-known phenomenon in experimental psychology in this context is the so-called “self-serving bias” (SSB), with the observation that healthy subjects tend to attribute more positive than negative characteristics to themselves [20,21]. Moreover, responses are faster in healthy subjects when attributing positive rather than negative self-referential personality traits to themselves, which is called the self-serving reaction time effect (SSRTE) [22,23,24]. Accordingly, in recall tasks, healthy subjects usually remember more positive than negative self-referential traits [25], which is referred to as the ‘mnemic neglect effect’ (MNE).

While SRP can be investigated in multiple different domains (e.g., in memory, emotional, language, spatial, auditory, or facial processing domains), a convergence for SRP across different domains has neuroanatomically been ascribed to cortical midline structures (CMS) [26], which overlap with core regions of the default-mode network (DMN). The CMS include the perigenual anterior cingulate cortex (PACC), the ventro- and dorsomedial prefrontal cortexes (VMPFC, DMPFC), the supragenual anterior cingulate cortex (SACC), the posterior cingulate cortex (PCC), as well as the medial parietal (MPC) and retrosplenial cortexes (RSC). Northoff et al. [26] furthermore suggested a functional subspecialization within the CMS. Based on cluster and factor metaanalyses of 27 functional imaging studies on self-related tasks, a functional suborganization into three regional clusters was suggested, distinguishing a ventral (comprising the MOFC, VMPFC, and PACC), a dorsal (comprising the DMPFC and SACC), and a posterior cluster (including the PCC, RSC, and MPC). These clusters were found to operate as process- rather than domain-specific. While the ventral cluster was linked to coding the degree of self-relatedness of intero- or exteroceptive stimuli, the dorsal cluster was related to the reappraisal and evaluation of self-directed stimuli (potentially embedded within the context of other non-self-related stimuli). Finally, the posterior cluster was found to be implicated in integrating self-referential stimuli within the temporal context and its degree of self-reference within autobiographical memory.

We investigated whether SRP and functional connectivity (FC) of CMS using resting-state fMRI would be altered in glioma patients compared to matched healthy controls. Self-related verbal information processing was investigated using a trait judgement and a trait recall task. Resting-state fMRI was applied in order to analyze the intrinsic FC of CMS to the DMN, as well as to the whole brain, under particular consideration of a potential functional specialization of CMS subregions with regard to self-referential verbal information processing.

## 2. Materials and Methods

### 2.1. Participants and Procedures

Nineteen patients with cerebral glioma (mean age: 43 ± 16 years, 12 males, 17 right-handed) and 19 age-matched (*p* = 0.971) healthy controls (mean age: 42 ± 15 years, 15 males, 18 right-handed) were included in the study. Only unilateral and histopathologically proven gliomas (according to Louis et al. [27]) were included, and the isodehydrogenase (IDH) mutation status was determined in each patient. Only patients ≥18 and <80 years of age with a Karnofsky index of ≥70 without motor or speech impairment participated in the study. Please note that the sample in the present study partially overlapped with the samples in previous publications [28,29], of which only those were included in the present study for whom self-referential testing was available in addition to a standardized neuropsychological examination and resting-state fMRI data. For the patients’ demographics and tumor characteristics, please see Table 1. All participants gave informed written consent. The study was approved by the local ethics committee of the Medical Faculty of the University of the RWTH Aachen (EK294-15) and conducted in accordance with the standards of Good Clinical Practice and the Declaration of Helsinki.

### 2.2. Neuropsychological Assessment

The neuropsychological assessment comprised the attention network task (ANT, [30]), the verbal learning and memory test (VLMT [31]), and the Rasch-based depression screening (DESC; [32]). For the specific scores from the complete neuropsychological testing of the present sample, please see Table 2. The current study mainly focused on the VLMT as a list-learning paradigm, which comprised eight trials with which verbal learning and recall were assessed. The relevant VLMT scores included the total learning, consolidation performance, and recognition scores.

### 2.3. Self-Referential Processing Tasks

To investigate the self-referential information processing and the SSE, SSRTE, and MNE, this study assessed the participants’ performance during two consecutive tasks, a trait judgment task and a trait recall task. The two tasks were performed using Presentation^®^ software (Version 19.0 11.14.16, Neurobehavioral Systems, Inc., Berkeley, CA, USA, www.neurobs.com). 

For the tasks, 50 trait adjectives (25 “positive” and 25 “negative”) were selected from the Aachen List of Trait Words (ALoT, [33]). Another 20 adjectives were chosen, and ten (5 positive and 5 negative each) of those were presented seamlessly in a training session before and another ten after the 50 test trials to exclude primacy and recency effects. Those trials were excluded from further analyses and are not reported here. Several stimuli properties, such as letter length, frequency of occurrence, valence, and social desirability, were controlled for in order to rule out systematic biases in choice and memory for positive versus negative stimuli [34,35].

#### 2.3.1. Trait Judgement Task

In the first task, the set of trait adjectives was randomly presented in white on a black screen for 2500 ms, intermitted by a variable fixation cross (between 500 and 800 ms). The participants had to indicate for each adjective if it was either self-describing or non-self-describing by pressing one of two buttons (yes-no) on a keyboard. The dichotomous coding was required in order to assess the memory effect in the subsequent task. The order of the fingers applied to the answer was fixed for right- and left-handed participants. The percentage of chosen positive and negative adjectives as self-describing and non-self-describing served as dependent variables to assess the SSE. Percentages were selected instead of absolute numbers to depict the proportions of the chosen adjectives, as the participants differed in the amounts of missed judgments. 

Additionally, choice-dependent reaction times (RT) were recorded. The choice dependent RT differences in the context of the SSE were further examined to assess the SSRTE.

#### 2.3.2. Trait Recall Task

In the second task, the participants had to recall as many of the previously presented adjectives as possible and had to repeat them orally in any order. For the completion of this task, the participants were given a fixed time interval of 5 min. The answers were voice-recorded (using Presentation^®^ software, Version 19.0 11.14.16, Neurobehavioral Systems, Inc., Berkeley, CA, USA, www.neurobs.com) and simultaneously written down by the experimenter.

The dependent variables used to assess the MNE were the percentages of recalled positive and negative self-describing and non-self-describing adjectives. Again, percentages were selected instead of absolute numbers to depict the proportions of recalled adjectives, as the participants differed in the amounts of missed judgments during the judgment task.

### 2.4. MRI Data Acquisition

An MRI examination was applied using a 3T Siemens Prisma MRI scanner equipped with a standard 20-channel head coil. The detailed scanning protocol was described in previous studies [29,36] and comprised the following pulse sequences: a sagittal 3D T1 magnetization-prepared rapid acquisition gradient echo sequence; a contrast-enhanced, T1-weighted turbo inversion recovery magnitude dark-fluid sequence; a T2-weighted TIRM dark-fluid scan, as well as a fluid attenuation inversion recovery sequence. In addition, RS-fMRI was implemented using echo planar imaging.

### 2.5. Preprocessing and FC Analyses

Functional preprocessing was performed using SPM12 as implemented in MATLAB 9.3 (Matlab, MA, USA). A detailed description of the image preprocessing protocol can be found in previous studies [29,36], and included semi-automatic tumor lesion segmentation using the ITK-SNAP software (masking T1 hypo- and T2-FLAIR hyperintensities for gliomas grade II-III, as well as T1 hypointensities and contrast-enhancing tumors for glioblastomas). The tumor volume was then calculated by counting the number of voxels in the tumor segmentation mask and corresponded to the volume in mm^3^. We divided it by 1000 to report the cm^3^ value for readability purposes. The functional images were realigned to the mean functional volume, unwarped, and co-registered to the structural T1-weighted image. The structural and functional images were normalized (including a binary tumor mask in case of patients’ data), and the functional images were smoothed with a 5 mm FWHM Gaussian kernel. The movement-related time series were regressed out with ICA-AROMA [37], and grand-mean scaling (mean-based intensity normalization), high-pass filtering (>0.01 Hz), as well as slice-timing correction were applied. 

For the FC analyses, the functional volumes were parcellated into a set of 50 predefined anatomical brain regions using the Brodmann areas (BA). Specific predefined regions, which are described below, were used as regions of interest (ROI), and each ROI’s mean time-series was extracted and the correlation to every other voxel in the brain mask was analyzed, resulting in a correlation map for every ROI. The correlation maps were Fisher z-transformed and used to compute the FC in the below analyses.

Firstly, the cortical midline structures (CMS) were parcellated into eight subregions with ROIs defined based on the study by Northoff et al. [26], which were grouped into 3 clusters, including cluster 1 (medial orbital prefrontal cortex (BA 11, 12), ventromedial prefrontal cortex (BA 10, 11), pre- and subgenual anterior cingulate cortexes (BA 24, 25, 32)), cluster 2 (supragenual anterior cingulate cortex (BA 24, 32), dorsomedial prefrontal cortex (BA 9)), and cluster 3 (medial parietal cortex (BA 7, 31), posterior cingulate cortex (BA 23), retrosplenial cortex (BA 26, 29, 30)). The FC within CMS was analyzed by computing the mean FC of the eight midline ROIs to one another, as well as by computing separate means for clusters 1, 2, and 3.

Secondly, a whole-brain mask was created, comprising all BA except for the eight CMS ROIs, and the mean FC of the CMS ROIs to the whole brain was computed. 

In the third step, lateral DMN ROIs were chosen according to Buckner et al. [38] and Pievani et al. [39], comprising the dorsolateral prefrontal cortex (BA 8), the inferior parietal lobule (BA 39, 40), the lateral temporal cortex (BA 21), and the hippocampal formation (BA 28, 34). The FC between the CMS ROIs and lateral DMN ROIs was analyzed. In addition, the FC of midline clusters 1, 2, and 3 and the lateral DMN ROIs of the ipsi- and contralesional hemispheres were computed. In the healthy controls, the mean FC of the left- and right-hemispheric DMN was computed and used as the baseline to compare the hemispheric connectivity between groups.

### 2.6. Statistics

The statistical analyses were performed using SPSS 27 (IBM Corp., Armonk, NY, USA) and R x64 3.4.2 (R Core Team, 2017, Vienna, Austria) [40].

#### 2.6.1. Behavioral Data

The neurobehavioral data were analyzed by applying independent sample *t*-tests (2-sided). For details please see Table 2, as these data were not the main focus of the study.

Regarding the analyses of the SSE, the SSRTE, and the MNE derived from the two behavioral tasks, it is important to note that in the analyses of RTs and the MNE, only those participants could be included who showed at least one choice in all conditions of the judgment task. Participants with missing values (i.e., empty cells) resulting from null choices had to be excluded from these analyses. The resulting N values are reported in Table 3 and Table 4. For all analyses of variance (ANOVAs), the partial eta-squared (*ƞ^2^_P_*) values are reported for the effect sizes with 90% confidence intervals (CI). Additionally, generalized eta-squared (*ƞ^2^_G_*) values are also reported as the effect sizes [41], which are recommended for repeated measures analyses by [42]. All reported post hoc analyses were Bonferroni-corrected. Therefore, only Bonferroni-corrected *p*-values are reported for the post hoc analyses. 

##### Trait Judgment Task—SSE and SSRTE

To assess the SSBE, 2 × 2 repeated measures analyses of variance (ANOVAs) with the valence of the adjectives (positive, negative) as the within-subject factor and the grouping condition (patients, controls) as the between-subject factor were calculated. The percentages of self-describing positive and negative adjectives and the percentages of non-self-describing positive and negative adjectives served as the dependent variables, respectively. 

The RTs were analyzed to assess the SSRTE, using 2 × 2 repeated measures ANOVAs with the valence of the adjectives (positive, negative) as the within-subject factor and the grouping condition (patients, controls) as the between-subject factor. The RTs of the self-describing positive and negative adjectives and the RTs of the non-self-describing positive and negative adjectives, respectively, were chosen as the dependent variables.

##### Trait Recall Task—MNE

A 2 × 2 × 2 repeated measures ANOVA with the valence of the adjectives (positive, negative) and the allocation of the adjectives (self-describing, non-self-describing) as the within-subject factors and the grouping condition (patients, controls) as the between-subject factor was calculated to inspect the MNE. The percentages of self-describing and non-self-describing positive and negative adjectives served as the dependent variables.

#### 2.6.2. MRI Data

##### FC within CMS

Group differences in the mean FC levels of the CMS were investigated by applying a univariate analysis of covariance (ANCOVA), including the mean FC as the dependent variable, the group (patients or controls) as the between-subject factor, and age as a covariate. Group differences in the FC levels of midline clusters 1, 2, and 3 were analyzed using a multivariate ANCOVA, including the FC levels of midline clusters 1, 2, and 3 as the dependent variables, the group (patients or controls) as between-subject factor, and age as a covariate.

##### FC of the CMS to the Whole Brain

The differences in the mean FC levels of the CMS ROIs to the whole brain between patients and controls were investigated by applying a univariate ANCOVA, including the mean FC of the CMS ROIs as the dependent variable, the group (patients or controls) as the between-subject factor, and age as a covariate. 

##### FC of the CMS to Lateral DMN Regions

The FC between CMS ROIs and lateral DMN ROIs was analyzed using a multivariate ANCOVA, including the FC levels between each of the eight midline ROIs to the lateral DMN as dependent variables, the group (patients or controls) as the between-subject factor, and age as a covariate. To explore whether the FC was altered in patients depending on the lesion site, the FC levels between midline clusters 1, 2, and 3 and the contra- and ipsilesional DMN ROIs were analyzed separately. The patients and controls were compared busing they means of two multivariate ANCOVAs, including the FC levels between clusters 1, 2, and 3 and the lateral DMN ROIs (first: cluster 1–3 to contralesional FC; second: cluster 1–3 to ipsilesional FC) as the dependent variables, the group (patients or controls) as the between-subject factor, and age as a covariate. 

#### 2.6.3. Correlation between FC, SSE, and MNE

The relationship between the FC, SSE, and MNE was tested separately for patients and controls using Pearson’s partial correlation analyses, controlling for effects of age. Please note that for the correlation analyses, the SSE and MNE were included, computed by the difference between the numbers of “chosen” positive and negative adjectives for the SSE and the difference between the numbers of recalled positive and negative adjectives for the MNE. The correlation analyses were two-side-tested with a significance level of *p* < 0.05 and Bonferroni-corrected for multiple testing, adjusted to *p* = 0.005. The tested variables included the mean FC levels of the CMS and midline clusters 1, 2, and 3. The partial correlations furthermore included the mean FC of the CMS to the whole brain, as well as to the lateral DMN regions. Finally, the correlation between the FC levels of midline clusters 1, 2, and 3; the lateral DMN ROIs (separately for the ipsi- and contralesional hemispheres); and the SSE and MNE parameters were tested.

## 3. Results

### 3.1. Behavioral Data

For detailed results regarding the neuropsychological assessment, please see Table 2. 

#### 3.1.1. Trait Judgment Task—SSE and SSRTE

**Figure 1:**
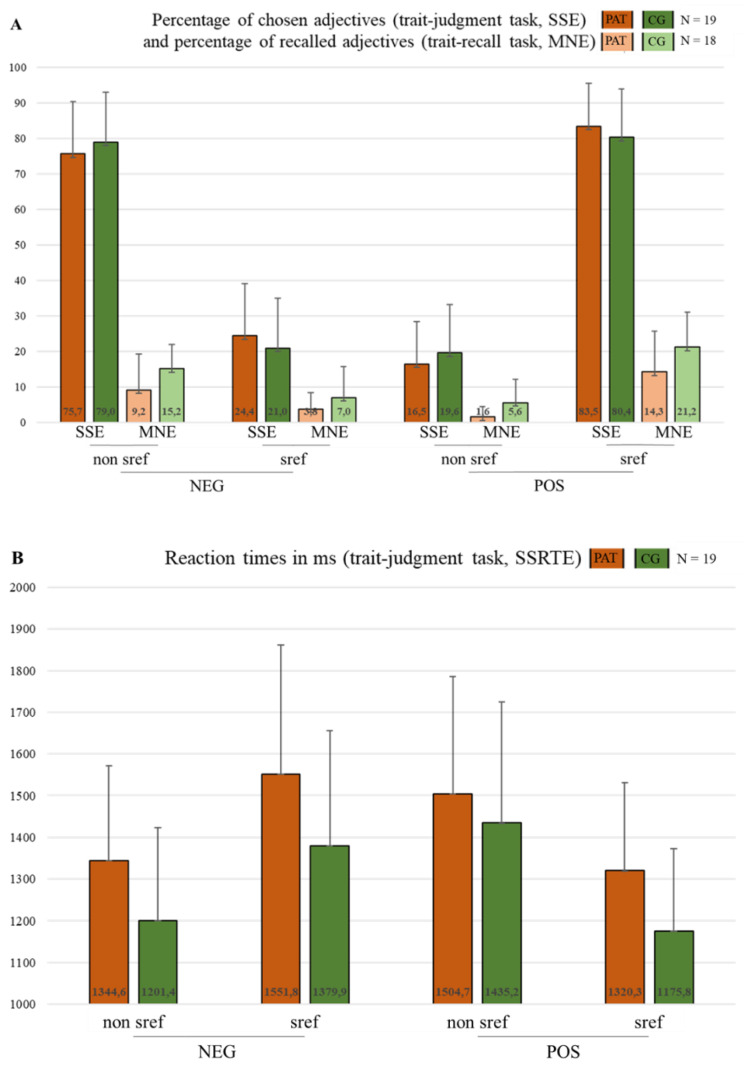
Trait judgment task (SSE) (**A**), SSRTE (**B**), and trait recall task (MNE) (**A**). The exact means and SD values are displayed in (**A**,**B**) (for the Bonferroni-corrected post hoc group differences, see Table 3 and Table 4). (**A**) **SSE** (2 × 2 repeated measures ANOVA): valence (pos sref > neg sref & neg non sref > pos non sref): *F*[1, 36] = 231.64, *p* < 0.001, *ƞ*^2^*_P_* = 0.87, 90% CI [0.79, 0.9], *ƞ*^2^*_G_* = 0.83; group (n.s.): *F*[1, 36] = 2.35, *p* = 0.13, *ƞ*^2^*_P_* = 0.06, 90% CI [0, 0.21], *ƞ*^2^*_G_* = 0.01; group*valence (n.s.): *F*[1, 36] = 0.001, *p* = 0.98, *ƞ*^2^*_P_* = 0.00002, 90% CI [0, 1], *ƞ*^2^*_G_* = 0.00001 (**A**)**—MNE** (2 × 2 × 2 repeated measures ANOVA): valence (n.s.): *F*[1, 34] = 3.67, *p* = 0.06, *ƞ*^2^*_P_* = 0.1, 90% CI [0, 0.26], *ƞ*^2^*_G_* = 0.01; group (CG > PAT): *F*[1, 34] = 7.68, *p* < 0.01, *ƞ*^2^*_P_* = 0.18, 90% CI [0.03, 0.36], *ƞ*^2^*_G_* = 0.09; group × valence (n.s.): *F*[1, 34] = 0.21, *p* = 0.65, *ƞ*^2^*_P_* = 0.01, 90% CI [0, 0.1], *ƞ*^2^*_G_* = 0.001; valence*choice (pos sref > neg sref & neg non sref > pos non sref): *F*[1, 34] = 53.86, *p* < 0.001, *ƞ*^2^*_P_* = 0. 61, 90% CI [0.42, 0.71], *ƞ*^2^*_G_* = 0.31; allocation (sref > non sref): *F*[1, 34] = 13.37, *p* < 0.001, *ƞ*^2^*_P_* = 0.28, 90% CI [0.08, 0.45], *ƞ*^2^*_G_* = 0.05; group*allocation (n.s.): *F*[1, 34] = 0.003, *p* = 0.96, *ƞ*^2^*_P_* = 0.0001, 90% CI [0, 1], *ƞ*^2^*_G_* = 0.00001; group*valence*allocation (n.s.): *F*[1, 34] = 0.98, *p* = 0.33, *ƞ*^2^*_P_* = 0.03, 90% CI [0, 0.16], *ƞ*^2^*_G_* = 0.01. (**B**) **SSRTE** (2 × 2 repeated measures ANOVA): valence (neg sref > pos sref: *F*[1,3 4] = 19.96, *p* < 0.001, *ƞ*^2^*_P_* = 0.37, 90% CI [0.16, 0.53], *ƞ*^2^*_G_* = 0.16; valence (pos non sref > neg non sref): *F*[1, 34] = 18.98, *p* < 0.001, *ƞ*^2^*_P_* = 0.36, 90% CI [0.15, 0.52], *ƞ*^2^*_G_* = 0.14; group (PAT sref > CG sref): *F*[1, 34] = 5.31, *p* < 0.05, *ƞ*^2^*_P_* = 0.14, 90% CI [0.01, 0.31], *ƞ*^2^*_G_* = 0.09; group (non sref n.s.: PAT = CG): *F*[1, 34] = 2.15, *p* = 0.15, *ƞ*^2^*_P_* = 0.06, 90% CI [0, 0.21], *ƞ*^2^*_G_* = 0.04; group*valence (sref n.s.): *F*[1, 34] = 0.08, *p* = 0.78, *ƞ*^2^*_P_* = 0.002, 90% CI [0, 0.08], *ƞ*^2^*_G_* = 0.001; group*valence (non sref n.s.): *F*[1, 34] = 0.66, *p* = 0.42, *ƞ*^2^*_P_* = 0.02, 90% CI [0, 0.14], *ƞ*^2^*_G_* = 0.01.

The ratings of the trait judgment task revealed the typical SSE, which was evident in higher choices of positive adjectives as self-describing compared to negative ones and a lower choice of positive adjectives as non-self-describing compared to negative ones (valence: *F*[1, 36] = 231.64, *p* < 0.001, *ƞ*^2^*_P_* = 0.87, 90% CI [0.79, 0.9], *ƞ*^2^*_G_* = 0.83), while no significant group difference (group: *F*[1, 36] = 2.35, *p* = 0.13, *ƞ*^2^*_P_* = 0.06, 90% CI [0, 0.21], *ƞ*^2^*_G_* = 0.01) and no significant interaction effect were found for both self-describing and non-self-describing choices (group*valence: *F*[1, 36] = 0.001, *p* = 0.98, *ƞ*^2^*_P_* = 0.00002, 90% CI [0, 1], *ƞ*^2^*_G_* = 0.00001; see Figure 1A). The exact means, SD values, and post hoc group differences can be found in Table 3.

Further, the SSRTE was evident in the shorter RT for positive compared to negative self-describing adjectives (valence: *F*[1, 34] = 19.96, *p* < 0.001, *ƞ*^2^*_P_* = 0.37, 90% CI [0.16, 0.53], *ƞ*^2^*_G_* = 0.16) and for negative compared to positive non-self-describing adjectives (valence: *F*[1, 34] = 18.98, *p* < 0.001, *ƞ*^2^*_P_* = 0.36, 90% CI [0.15, 0.52], *ƞ*^2^*_G_* = 0.14). Group differences were only found for self-describing choices, whereby the control subjects’ RT values were shorter compared to the patients RT values (group: *F*[1, 34] = 5.31, *p* < 0.05, *ƞ*^2^*_P_* = 0.14, 90% CI [0.01, 0.31], *ƞ*^2^*_G_* = 0.09). With regard to the non-self-describing choices, the patients and controls did not differ (group: *F*[1, 34] = 2.15, *p* = 0.15, *ƞ*^2^*_P_* = 0.06, 90% CI [0, 0.21], *ƞ*^2^*_G_* = 0.04). Moreover, no significant interaction effect was found for self-describing (interaction: *F*[1,34] = 0.08, *p* = 0.78, *ƞ*^2^*_P_* = 0.002, 90% CI [0, 0.08], *ƞ*^2^*_G_* = 0.001) and non-self-describing choices (interaction: *F*[1, 34] = 0.66, *p* = 0.42, *ƞ*^2^*_P_* = 0.02, 90% CI [0, 0.14], *ƞ*^2^*_G_* = 0.01). (For the results, see Figure 1A,B; for the exact means, SD values, and post hoc group differences, please see Table 3.)

**Table 3:** Choices and reaction times in the trait judgment task.

Total Numbers and Percentages of Chosen Adjectives (Trait Judgment Task)										
							Groups			
							Patients (*N* = 19)		Controls (*N* = 19)	
							Valence			
							POS ^a^	NEG ^a^	POS	NEG
Allocation	Group	Valence	Mean (total)	SD (total)	Mean (%)	SD (%)	*p* ^1^	*p*	*p*	*p*
self-describing	Patients	POS	20.2	3.5	83.5	12.0				
		NEG	6.0	3.6	24.4	14.7				
	Controls	POS	20.0	3.4	80.4	13.6				
		NEG	5.2	3.5	21.0	14.1				
non-self-describing	Patients	POS	4.0	2.9	16.5	12.0		<0.001	1	<0.001
		NEG	18.6	3.6	75.7	14.7			<0.001	1
	Controls	POS	4.9	3.4	19.6	13.6				<0.001
		NEG	19.4	3.6	79.0	14.1				
**Reaction times in ms (trait judgment task)**										
self-describing	Patients	POS	1320.3	211.2				0.08	0.25	1
		NEG	1551.8	309.2					<0.001	0.52
	Controls	POS	1175.8	197.5						0.09
		NEG	1379.9	275.8						
non-self-describing	Patients	POS	1504.7	280.5				0.42	1	<0.01
		NEG	1344.6	227.6					1	0.20
	Controls	POS	1435.2	289.1						<0.05
		NEG	1201.4	221.4						

Total numbers and percentages of the chosen adjectives, RTs, and results from the post hoc analyses of the SSE and SSRTE in the trait judgment task.^1^ Bonferroni-corrected *p*-values; ^a^ POS: positive; NEG: negative; SD = standard deviation.

#### 3.1.2. Trait Recall Task—MNE

Concerning the trait recall task, the self-serving MNE was reflected in the enhanced recall of positive self-describing adjectives and negative non-self-describing adjectives compared to negative self-describing and positive non-self-describing adjectives (valence*choice: *F*[1, 34] = 53.86, *p* < 0.001, *ƞ*^2^*_P_* = 0. 61, 90% CI [0.42, 0.71], *ƞ*^2^*_G_* = 0.31), i.e., a “self-serving” effect in both groups. Generally, the control subjects showed better recall performance in comparison to the patients (group: *F*[1, 34] = 7.68, *p* < 0.01, *ƞ*^2^*_P_* = 0.18, 90% CI [0.03, 0.36], *ƞ*^2^*_G_* = 0.09). Further, a significant main effect was found for the overall allocation of the adjectives, i.e., self-describing adjectives were generally better recalled compared to non-self-describing adjectives (allocation: *F*[1, 34] = 13.37, *p* < 0.001, *ƞ*^2^*_P_* = 0.28, 90% CI [0.08, 0.45], *ƞ*^2^*_G_* = 0.05). There was no significant main effect for the valence (valence: *F*[1, 34] = 3.67, *p* = 0.06, *ƞ*^2^*_P_* = 0.1, 90% CI [0, 0.26], *ƞ*^2^*_G_* = 0.01) and no significant interaction effect between the valences of the adjectives and groups (group*valence: *F*[1, 34] = 0.21, *p* = 0.65, *ƞ*^2^*_P_* = 0.01, 90% CI [0, 0.1], *ƞ*^2^*_G_* = 0.001), nor between the allocation values of the adjectives and groups (group × allocation: *F*[1, 34] = 0.003, *p* = 0.96, *ƞ*^2^*_P_* = 0.0001, 90% CI [0, 1], *ƞ*^2^*_G_* = 0.00001). Further, there was no triple interaction between the valences of the adjectives or the allocation values of the adjectives and groups (group × valence × allocation: *F*[1, 34] = 0.98, *p* = 0.33, *ƞ*^2^*_P_* = 0.03, 90% CI [0, 0.16], *ƞ*^2^*_G_* = 0.01). (For the results, please see Figure 1A; the exact means, SD values, and post hoc group differences can be found in Table 4.)

**Table 4:** Summary of the recall performances in the trait recall task.

Total Numbers and Percentages of Recalled Adjectives (Trait Recall Task)														
							Group							
							Patients (*N* = 18)				Controls (*N* = 18)			
							Allocation							
							Self-Describing		Non-Self-Describing		Self-Describing		Non-Self-Describing	
							Valence							
							POS ^a^	NEG ^a^	POS	NEG	POS	NEG	POS	NEG
Groups	Allocation	Valence	Mean (total)	SD (total)	Mean (%)	SD (%)	*p* ^1^	*p*	*p*	*p*	*p*	*p*	*p*	*p*
Patients	self-describing	POS	3.5	2.9	14.3	11.4					1	1	0.22	1
		NEG	0.9	1.2	3.8	4.7	<0.05				<0.001	1	1	<0.001
	non-self-describing	POS	0.4	0.7	1.6	2.9	<0.01	2.66			<0.001	0.49	0.62	<0.001
		NEG	2.3	2.5	9.2	10.1	4.71	1.26	0.11		<0.05	1	1	1
Controls	self-describing	POS	5.3	2.5	21.2	9.9								
		NEG	1.7	2.2	7.0	8.7					<0.01			
	non-self-describing	POS	1.4	1.6	5.6	6.5					<0.001	1		
		NEG	3.7	1.7	15.2	6.8					1	0.1	<0.01	

Total numbers and percentages of recalled adjectives and post hoc analyses for the MNE in the trait recall task. ^1^ Bonferroni-corrected *p*-values. ^a^ POS: positive; NEG: negative; SD = standard deviation.

### 3.2. Results fMRI Data

#### 3.2.1. FC within CMS

The univariate ANCOVA did not reveal significant differences in the mean FC values of the CMS between the patients and controls (*F*[1, 35] = 0.056, *p* = 0.814). Accordingly, the FC values of midline clusters 1–3 did not differ between groups, as revealed by the multivariate ANCOVA (*F*[3, 33] = 0.1.482, *p* = 0.243).

#### 3.2.2. FC of the CMS to the Whole Brain

**Figure 2:**
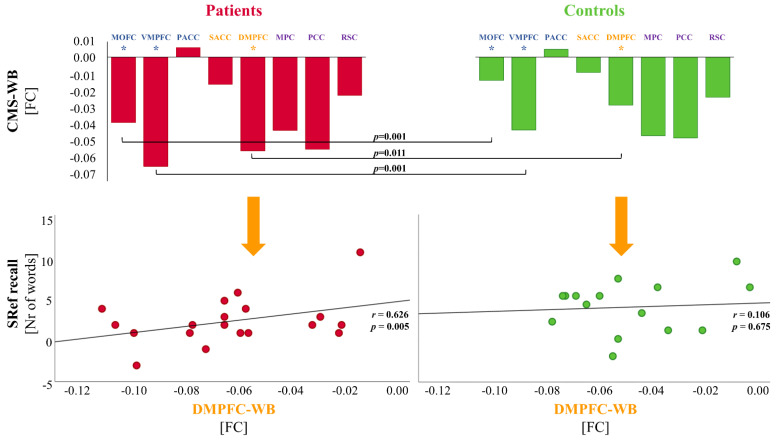
Correlations between the FC, SSE, MNE in patients and controls. *Upper row:* Resting-state fMRI connectivity values for each of the eight cortical midline structures (CMS) subregions (MOFC: medial orbitofrontal cortex; VMPFC: ventromedial prefrontal cortex; PACC: perigenual anterior cingulate cortex; SACC: supragenual anterior cingulate cortex; DMPFC: dorsomedial prefrontal cortex; MPC: medial parietal cortex; PCC: posterior cingulate cortex; RSC: retrosplenical cortex) to the whole brain. The colors indicate clustering into the ventral (blue), dorsal (orange), and posterior (purple) clusters. Significant between-group differences are indicated by asterisks (*p* < 0.05). *Lower row:* Significant correlations of self-referential recall test metrics for the numbers of words with the functional connectivity of the DMPFC to the whole brain were found in patients but not in controls (two-side-tested with a significance level of *p* < 0.05 and Bonferroni-corrected for multiple testing, adjusted to *p* = 0.005).

The patients and controls differed significantly in the mean FC values of the CMS to the whole brain, as shown by the univariate ANCOVA results (*F*[1, 35] = 6.786, *p* = 0.013). The patients had stronger negative FC (anti-correlation) between CMS to the whole brain than the controls. To identify which CMS subregion was decisive for this difference, a multivariate ANCOVA including the FC values of each of the eight midline ROIs to the whole brain was performed post hoc. The model was significant (*F*[8, 26] = 2.775, *p* = 0.021), indicating significant group differences in the FC values of the medial prefrontal cortex comprising the medial orbital prefrontal cortex (MOFC), ventromedial prefrontal cortex (VMPFC), and dorsomedial prefrontal cortex (DMPFC) to the whole brain (*F*[1, 35] = 13.448, *p* = 0.001, (*F*[1, 35] = 12.376, *p* = 0.001), and (*F*[1, 35] = 7.184, *p* = 0.011), respectively). Each of these three midline ROIs showed higher anti-correlations to the whole brain in the patients as compared to the controls (please see Figure 2).

#### 3.2.3. FC of the CMS to Lateral DMN Regions

The multivariate ANCOVA results revealed no significant group difference in the FC values between the CMS and lateral DMN (*F*[8, 28] = 0.641, *p* = 0.737). Similarly, no differences in the FC of values midline clusters 1–3 to any of the lateral DMN ROIs were found between the patients and controls (*F*[12, 24] = 1.585, *p* = 0.204), and no significant differences were observed for the lesioned and non-lesioned hemispheres compared to the controls.

### 3.3. Correlations between the FC, SSE, and MNE

The partial correlation analyses of the mean FC values of the CMS; midline clusters 1–3; or of the CMS to the whole brain with the SSE and MNE did not reveal significant results, either for the patients or the controls. Similarly, the partial correlation analyses of the mean FC values of the CMS to lateral DMN regions, as well as the FC values of midline clusters 1–3 to the contra- or ipsilesional hemispheric DMN ROIs, did not show significant correlations with the SSE and MNE parameters.

Based on the significant group differences in FC values between specific CMS subregions (MOFC, VMPFC, DMPFC) to the whole brain, the partial correlations for the FC levels of each of these CMS subregions to the whole brain and the SSE and MNE were additionally computed (two-side-tested with a significance level of *p* < 0.05 and Bonferroni-corrected for multiple testing, adjusted to *p* = 0.005). The results showed a positive correlation between the MNE and FC of the DMPFC to the whole brain FC (*r* = 0.626, *p* = 0.005, respectively) in patients, but not in controls (please see Figure 2).

## 4. Discussion

In view of the chronically progressive structural disintegration of the brain as encountered in diffusely infiltrating glioma, it is unclear if and depending on which neural substrates the lesioned brain is able to maintain an adequate sense of self. We investigated self-related verbal information processing in glioma patients using a trait judgement task and a trait recall task, as well as resting-state FC of CMS as core regions of the DMN, which are regarded as essential for self-mentalizing processes. Resting-state FC within the CMS as well as ofCMS to the DMN and to whole-brain were compared between patients and healthy controls. Even though glioma patients showed significant cognitive impairment, especially with regard to verbal memory functions, their metacognitive abilities were found to be preserved, with both the SSE as well as the MNE being maintained in patients. While the intrinsic FC levels within CMS as well as of CMS to the DMN did not differ across groups, FC between the medial prefrontal cortex and whole-brain was significantly decreased in glioma patients. FC specifically of the DMPFC was found to be associated with the MNE in patients.

### 4.1. Cognitive Dysfunction and Self-Referential Processing in Glioma Patients

Complying with the known literature [1,2,3,4,5,6], the glioma patients in our study showed preoperatively significantcognitive dysfunction. Namely, verbal learning and verbal recall were significantly impaired, as well as attentional deficits were observed. Patients furthermore showed significantly higher depression scores as compared to controls. Although it had previously been reported that affective disorders and depression may interfere with the self-serving bias [43], our patients appeared to be unaffected in this regard. A potential bias affecting neurocognitive functions and functional connectivity measures in patients might be caused by differing tumor and edema volumes, although previous studies [44,45] have shown, that cognitive dysfunction and functional connectivity do not necessarily correlate with tumor lesion volumes. Considering that patients and controls differed in levels of education (while being matched for age and handedness) in our sample, it cannot be excluded that between-group differences in cognitive functions might have been confounded by educational differences (although group difference comprised only 2 years, with all patients having finished at least secondary school, so that a major impact on test metrics was not to be expected). Even more remarkably, the patients were found not to differ in their metacognitive abilities compared to controls, despite educational and cognitive differences, as the patients also consistently showed the SSE in the trait judgment task, with higher choices of positive adjectives as self-describing and lower choices of positive adjectives as non-self-describing. Likewise, shorter RTs for positive compared to negative self-describing adjectives and for negative compared to positive non-self-describing adjectives were observed, indicating the SSRTE in both groups. In total, patients recalled less adjectives than controls, corresponding to the observed deficits in verbal memory functions. Remarkably, the MNE was maintained, despite the occurrence of verbal memory deficits, with enhanced recall of positive self-describing adjectives and negative non-self-describing adjectives in both patients and controls. These findings suggest that the verbal recall of self-related information seems to be more robustly preserved in the lesioned brain as compared to purely semantic verbal memory retrieval, suggesting distinct neural substrates for self-related verbal information processing, as supported by previous fMRI findings [46] showing SRP in the verbal domain to be neurally dissociable from other forms of semantic processing.

### 4.2. DMN and Self-Referential Processing

Multiple studies [46,47,48,49,50,51,52,53] have linked the DMN to self-mentalizing abilities, which is why we investigated FC values of CMS specifically to the lateral DMN in patients and controls. Emanating from observations in early functional imaging studies of functional activity of DMN regions, mainly in the resting condition [54,55,56], the DMN has been related to mind wandering, introspection, and self-relatedness [46,47]. In accordance with this notion, fMRI studies on verbal SRP [57] reliably showed involvement of the medial PFC (including the DMPFC, anterior or middle MPFC, VMPFC), as well as of medial parietal cortex (including the PCC, precuneus, inferior parietal lobe, and posterior temporoparietal junction) and anterior medial temporal cortex (including the insula, amygdala, and hippocampus). With the ambiguity of the DMN being deactivated during most active tasks while being activated in the resting condition, fMRI studies investigating task-driven verbal SRP activations confirmed a large overlap with those regions that are active during the resting-state [57]. Overlapping responses have in particular been described in the middle MPFC, the posterior ACC, the ventral PCC, and the border of the ventral precuneus [56]. On the other hand, greater responses in verbal SRP as compared to resting-state activity have been reported within the middle MPFC [58], while higher activity in the resting state was described within insular cortex and right temporoparietal junction or inferior parietal lobe. The DMN regions during the resting state, thus, may largely—although not completely—overlap with task-driven involvement of SRP in the DMN structures. While intrinsic FC levels of CMS within the DMN did not differ between patients and controls in our study, no significant correlations were found between the SSE or the MNE and resting-state FC of the DMN in either group, which might have been due to the relative incongruencies between task-driven SRP activations and the intrinsic FC of the DMN in the resting-state. Moreover, referencing the conceptual framework used by James [59], Frewen et al. [57] further differentiated mind-related “semantic” or “verbal SRP” (V-SRP) from a bodily sense of self with “somatic” or “non-verbal SRP” (NV-SRP), with the latter being believed to be more prevalent in the resting condition. Correlating verbal SRP performance in our study with “somatic SRP” resting-state DMN representations may therefore has failed to show significant associations. Comparing V-SRP- and NV-SRP-related fMRI activations, Araujo et al. [60] described stronger responses in NV-SRP for the bilateral insula, medial superior parietal lobe (M-SPL), and bilateral anterior temporal parietal function, whereas the V-SRP was found to be more strongly associated with the medial prefrontal cortex (MPFC), posterior cingulate, and precuneus. Strong reciprocal interactions are assumed between both SRP modalities, and bottom-up as well as top-down processes are believed to have modulatory effects on both [57]. Notably, the DMPFC has previously been identified as a common core region for both V-SRP as well as NV-SRP [60], which may suggest a supramodal role of the DMPFC for integrating semantic and somatic self-referential information processes.

### 4.3. CMS as Convergence Zone for Self-Referential Processing

With self-referential processing occurring in multiple domains (e.g., verbal, visual, sensory, spatial, emotional), various domain-specific networks might be involved, depending on the task. However, based on a meta-analysis of different PET and fMRI studies on self-related tasks, Northoff et al. [26] suggested CMS as the essential convergence zone for self-referential processing across different domains. With CMS being anatomically densely interconnected to subcortical midline regions, the CMS have been suggested to subserve both bottom-up as well as top-down modulatory effects on sensory, self-referential, and higher-order processing [26]. As such, CMS were proposed as core regions for self-mentalizing processes, which is supported by several other studies [12,61,62,63,64]. While the CMS are considered as functional unit, a functional subspecialization has been suggested within the CMS, which has been described to be process- rather than domain-specific [26]. We therefore subparcellated the CMS in our study into eight subregions, as previously suggested by Northoff et al. [26], in order to investigate whether CMS subregions would differ with regard to the intrinsic FC levels across groups, and whether they would be differentially associated with the SSE and the MNE. Interestingly, the intrinsic FC levels did neither differ within the CMS nor between CMS to lateral DMN regions when comparing both groups. It should be noted, that the present study specifically focused on FC of CMS to lateral DMN regions, and does not contradict previously described differences in mean hemispheric DMN connectivity levels across groups [29]. In patients, however, intrinsic FC levels of CMS were found to be significantly decreased specifically between the medial prefrontal cortex and whole-brain in the sense of stronger anticorrelations as compared to the controls. This might be ascribed to tumor-lesion-induced disconnections of lateral brain regions from the CMS depending on the tumor location, although an additional analysis showed, that FC of CMS to whole- brain was not significantly reduced (*F*[8, 9] = 0.772, *p* = 0.637) in frontal tumors (*n* = 9) when compared to non-frontal tumors (*n* = 10). This decrease in intrinsic FC between the medial prefrontal cortex and whole-brain, with notably unaltered connectivity between medial prefrontal cortex and lateral DMN regions, may relate to the current notion of regarding the CMS and the DMN as neural core substrates of self-related information processing, although this association cannot be proven by the present data. Interestingly though, FC specifically of the dorsomedial prefrontal cortex (DMPFC) to whole-brain was found to be associated with the MNE in our patients, which may indicate a modulatory role of the DMPFC in the lesioned brain for self-related verbal information processing in the memory domain.

### 4.4. DMPFC in Self-Referential Processing and Memory

The association of FC of the DMPFC to whole-brain with the MNE in our patients complies well with previous findings, in that the medial prefrontal cortex has repeatedly been reported to be involved both in memory encoding and retrieval, as well as SRP. By showing that SRP is functionally dissociable from other forms of semantic processing, Kelley et al. [46] previously demonstrated the MPFC to be selectively engaged during SRP, which was suggested to provide a neural substrate for self-bias in memory. In the study by Macrae et al. [65], MPFC activity was reported to predict the subsequent memory performance and judgements of self-relevance. Considered that the MPFC has mostly been described as showing decreased activation in cognitive tasks [56], with continuous activity during self-related information processing [65,66], the MPFC has been regarded as mediating the memory advantage in SRP, and has been identified as a core modulatory unit within the DMN [53]. In line with this notion, lesion studies showed that damage to the MPFC abolishes the SSE [67]. Differentiating distinct cognitive and affective components of the self, the MPFC has been linked to the self-relevance of information, while emotional valence has been related to the ventral ACC [68]. Using a trait judgement task in a task-related fMRI study, Macrae et al. [65] found the DMPFC to be involved both in retrieval performance, as well as in judging self-relevance. The DMPFC has furthermore repeatedly been reported to be engaged in autobiographical memory retrieval [69,70]. Using the PET-TMS and another task in which personality trait adjectives had to be episodically retrieved for oneself or others, Lou et al. [71] found the DMPFC to interact with the posterior CMS, as well as with the lateral brain regions, so that the MPFC was previously suggested to be a nodal core region, specifically modulating the episodic retrieval of self-related verbal information processing via interactions with distributed brain regions.

This understanding of the DMPFC as an important hub region for integrating complex higher order functions is strongly supported by recent findings ofthe functional and anatomical segregation of the DMPFC [72] into four distinct clusters, distinguishing a rostrodorsal, a rostroventral, and right and left caudal clusters. Additionally, the rostral clusters of the DMPFC have specifically been linked to the amygdala and hippocampus, with associated memory and social cognitive functions, while the rostroventral cluster showed the strongest connectivity to the DMN. The right caudal cluster was reported to be strongly connected to the frontoparietal network, while the left caudal cluster was strongly connected to the salience network. These findings provide a neuroanatomical basis for assumed interactions of the DMPFC across different large-scale distributed networks, and for its potential involvement in, if not orchestration of, complex higher order cognitive processes in the context of SRP and memory functions. The left DMPFC (besides the VLPFC) has previously been found to be specifically engaged in mnemonic context retrieval [73]. With the DMPFC also being engaged in the frontoparietal control network [66], it is furthermore believed to integrate cognitive control processes into SRP and affect regulation [74]. While the VMPFC has predominantly been linked to bottom-up affective processing [57], the DMPFC has rather been related to top-down control, as well as to the meta-cognitive processing of social and emotional experiences, presumably mediating a greater sense of detachment and a more allocentric or observer perspective, even towards ’the self’ [75,76]. This notion further complies with the observed involvement of the DMPFC in social perspective-taking paradigms such as the theory of mind [75,76,77], which is why the DMPFC is regarded as the interface between neural networks subserving internally and externally directed cognition [78].

This “multimodality” of the DMPFC may provide the grounds in our patients to maintain the MNE despite significant impairments in verbal memory functions, potentially by interacting with supportive distributed brain regions beyond the DMN, which further extends our recent understanding of the dynamic rather than localizational organizational structure of the neural network representations of the self [79].

### 4.5. Limitations

As we analyzed resting-state fMRI data, it was not possible to investigate immediate self-referential task-related neural activity in our study. Using resting-state fMRI data only allowed us to give a rough estimate of the basic intrinsic functional architecture of CMS in relation to lateral brain regions. Using empiric ROIs in our study to investigate intrinsic FC within the DMN might be regarded as a further shortcoming, as it neglects the potentially tumor-induced reorganization and spatiotemporal redistribution of DMN representations in individual patients, as well as our recent understanding of a more elaborate functional suborganization, with dynamic and context-dependent rather than static neural representations of the DMN [80]. We however used this simplified model in our study due to the heterogeinity of tumor lesions and the small sample size, which were the major limitation of the present study, in order to be able to compare the corresponding regions across groups. Remarkably, the connectivity of CMS within the DMN proved to be surprisingly stable in our patients, despite substantial structural damage and different tumor locations. We are, however, aware of the mere exploratory character of the present study, which needs to be validated and further refined in larger and possibly multicenter studies, with more homogeneous patient samples. From the clinical perspective, the preservation of metacognitive abilities in brain tumor treatment is presently mostly neglected in clinical routines, although it would be highly desirable to be taken more into consideration for individualized tumor treatment concepts. Therefore, improving the understanding and elucidating the neural substrates of metacognitive processes in the human brain should be the subject of future studies, in order to provide the grounds for improved treatment concepts aiming not onlyat preservation of basic somatosensory or cognitive functions, but also at preservation of metacognitive abilities and the socioaffective integrity of the self.

## 5. Conclusions

Despite significant cognitive impairment, glioma patients showed preserved metacognitive abilities, as well as preserved intrinsic connectivity of CMS within the DMN. Future studies with larger and more homogenous patient samples are needed to further elucidate, whether preserving the functional integrity of CMS in therapeutic interventions might be essential for maintaining self-related verbal information processing in glioma patients.

## Figures and Tables

**Table 1:** Clinical description of the included patients.

Pat IDH		Side	Grade	Location	Diagnosis	Vol. ^c^ (cm^3^)	Age (Years)	Educ. ^e^ (Years)
1	y	r	II	Temporal	Astrocytoma	30	30–35	13
2	y	r	II	Frontal	Oligodendro-glioma	2	36–40	13
3	y	r	III	Frontal	Anaplastic astrocytoma ^a^	30	36–40	16
4	y	r	III	Frontal	Anaplastic astrocytoma	155	30–35	15
5	y	r	III	Frontal	Anaplastic oligodendro-glioma	96	30–35	18
6	y	l	II	Temporo-insular	Astrocytoma	32	30–35	15
7	y	l	II	Frontal	Astrocytoma	158	26–30	13
8	y	l	II	Frontal	Astrocytoma	51	26–30	16
9	y	l	II	Frontal	Oligodendro-glioma	22	26–30	13
10	y	l	II	Parietal	Astrocytoma	64	55–60	18
11	y	l	III	Frontal	Anaplastic astrocytoma	21	50–55	13
12	y	l	III	Frontal	Anaplastic astrocytoma	49	40–45	13
13	y	l	III	Parietal	Anaplastic astrocytoma	119	20–25	13
14	y	l	III	Parietal	Anaplastic astrocytoma	114	40–45	13
15	n	l	I	Hippocampal	Dysembryoplastic neuroepithelial tumor	24	40–45	15
16	n	l	IV	Temporo-parietal	Glioblastoma multiforme ^b^	13/50 ^d^	50–55	13
17	n	l	IV	Fronto-parietal	Glioblastoma multiforme	11/44 ^d^	76–80	13
18	n	l	IV	Frontal	Anaplastic astrocytoma/Glioblastoma multiforme	11/22 ^d^	60–65	15
19	n	bi	IV	Callosum	Glioblastoma multiforme	55/5 ^d^	70–75	9

Pat = patient; IDH = isocitrate dehydrogenase; y = yes; n = no; l = left; r = right; bi = bilateral. ^a^ Recurrent anaplastic astrocytoma after first tumor resection and adjuvant radiochemotherapy. ^b^ Recurrent glioblastoma after first tumor resection and adjuvant radiochemotherapy. ^c^ Vol. = tumor volume; ^d^ peritumoral edema; ^e^ years of education were computed by the sum of years spent in the school career and further training/study.

**Table 2:** Sociodemographic and neuropsychological test data.

	Patients N = 19		Controls N = 19		Sig. *	ES ^1^	CI ^2^	
Variable	Mean	SD	Mean	SD	*p*		Lo	Up
Age (years)	42.6	16.4	42.4	14.9	0.97	0.01	−0.62	0.65
Educ. (years) ^a^	14.1	2.1	16.3	2.2	**0.00**	−1.00	−1.68	−0.33
DESC ^b^	6.5	5.9	3.3	2.4	**0.04**	0.70	0.04	1.35
VLMT_acqu_^c^	48.4	12.3	58.4	7.0	**0.00**	−0.98	−1.65	−0.31
VLMT_consol_	2.21	2.4	0.58	1.5	**0.02**	0.81	0.15	1.47
VLMT_recog_	12.1	3.1	13.8	1.6	**0.03**	−0.70	−1.35	−0.04
ANT__Rges_^d^	281.5	12.6	284.5	2.9	0.32	−0.32	−0.96	0.32
ANT__RT_Rges_	608.6	198.1	505.1	63.7	**0.04**	0.7	0.03	1.34
ANT__RT_conflict_	41.8	34.7	29.1	21.5	0.18	0.43	−0.21	1.07
ANT__RT_Orient_	14.5	23.6	18.3	12.6	0.54	−0.20	−0.83	0.44
ANT__RT_Alarm_	93.4	34.3	75.8	23.8	0.07	0.58	−0.07	1.23
ANT__error_	6.5	12.6	3.5	2.9	0.32	0.32	−0.32	0.96

^a^ Years of education were computed by the sum of years spent in the school career and further training/study; ^b^ DESC = Rasch-based depression screening (DESC; [32]); ^c^ VLMT = verbal learning and memory test (VLMT; [31], VLMT_acqu_ = acquisition, VLMT_consol_ = consolidation, VLMT_recog_ = recognition; ^d^ ANT = attention network test (ANT; [30]), ANT__Rges_ = total of performed trials, ANT__RT_Rges_ = reaction time of total performed trials, ANT__RT_conflict_ = conflict effect, ANT__RT_Orient_ = orientation effect, ANT__RT_Alarm_ = alarm effect, ANT__error_ = total number of errors; SD = standard deviation; * *p*-value for mean difference (2-tailed *t*-Test), significant values are indicated in **bold**; ^1^ ES = Hedges bias corrected effect size; ^2^ CI = confidence interval for effect size, Lo = lower bound, Up = upper bound.

## Data Availability

Due to ethical considerations and privacy of the study participants, the data are not publicly available but can be obtained upon request from the corresponding author.
